# Medication overuse headache is a manifestation of opioid induced hyperalgesia: a neuroimmune hypothesis and novel approach to treatment

**DOI:** 10.1186/1129-2377-14-S1-P162

**Published:** 2013-02-21

**Authors:** P Rolan, JL Johnson

**Affiliations:** 1 University of Adelaide, Australia

## Introduction

Patients with chronic headache who consume large amounts of analgesics are often encountered in clinical practice. Excessive intake of analgesics is now considered to be a cause, rather than simply a consequence of frequent headaches, and as such the diagnosis “medication overuse headache” has been formulated[1]. Despite the prevalence and clinical impact of medication overuse headache the pathophysiology behind this disorder remains unclear and specific mechanism-based treatment options are lacking.

## Methods

Preclinical and clinical data from the literature were reviewed.

## Results and discussion

Although most acute headache treatments have been alleged to cause medication overuse headache, here we conclude that opioids are the drug class most strongly associated with worsening headache. Recent evidence indicates that chronic opioid administration may exacerbate pain in the long-term by non-specifically activating Toll-Like Receptor-4 on glial cells, resulting in a pro-inflammatory state that manifests clinically as hyperalgesia[2]. We hypothesise that medication overuse headache is a specific form of opioid-induced hyperalgesia, which derives from a cumulative interaction between central sensitisation, due to repeated activation of nociceptive pathways by recurrent headaches, and pain facilitation due to glial activation.

## Conclusions

Treatment strategies directed at inhibiting glial activation may be of benefit in the management of medication overuse headache. Potential treatment options could include agents such as ibudilast, minocycline and (+)-naltrexone.
